# Patients’ Perspectives on Qualitative Olfactory Dysfunction: Thematic Analysis of Social Media Posts

**DOI:** 10.2196/29086

**Published:** 2021-12-14

**Authors:** Jane K Parker, Christine E Kelly, Barry C Smith, Aidan F Kirkwood, Claire Hopkins, Simon Gane

**Affiliations:** 1 Department of Food and Nutritional Sciences School of Chemistry, Food and Pharmacy University of Reading Reading United Kingdom; 2 AbScent Andover United Kingdom; 3 Centre for the Study of the Senses Institute of Philosophy School of Advanced Study, University of London London United Kingdom; 4 Ear, Nose and Throat Department Guy’s and St Thomas’ Hospitals London United Kingdom; 5 Royal National Ear, Nose and Throat and Eastman Dental Hospitals London United Kingdom

**Keywords:** olfactory dysfunction, parosmia, phantosmia, olfactory perseveration, trigger foods, mental health, COVID-19, patients’ perspective, thematic analysis, social media, perspective, smell, nose, symptom, concern, support

## Abstract

**Background:**

The impact of qualitative olfactory disorders is underestimated. Parosmia, the distorted perception of familiar odors, and phantosmia, the experience of odors in the absence of a stimulus, can arise following postinfectious anosmia, and the incidences of both have increased substantially since the outbreak of COVID-19.

**Objective:**

The aims of this study are to explore the symptoms and sequalae of postinfectious olfactory dysfunction syndrome using unstructured and unsolicited threads from social media, and to articulate the perspectives and concerns of patients affected by these debilitating olfactory disorders.

**Methods:**

A thematic analysis and content analysis of posts in the AbScent Parosmia and Phantosmia Support group on Facebook was conducted between June and December 2020.

**Results:**

In this paper, we identify a novel symptom, olfactory perseveration, which is a triggered, identifiable, and usually unpleasant olfactory percept that persists in the absence of an ongoing stimulus. We also observe fluctuations in the intensity and duration of symptoms of parosmia, phantosmia, and olfactory perseveration. In addition, we identify a group of the most common items (coffee, meat, onion, and toothpaste) that trigger distortions; however, people have difficulty describing these distortions, using words associated with disgust and revulsion. The emotional aspect of living with qualitative olfactory dysfunction was evident and highlighted the detrimental impact on mental health.

**Conclusions:**

Qualitative and unsolicited data acquired from social media has provided useful insights into the patient experience of parosmia and phantosmia, which can inform rehabilitation strategies and ongoing research into understanding the molecular triggers associated with parosmic distortions and research into patient benefit.

## Introduction

### Background: Olfactory Dysfunction Before and Since COVID-19

Until recently, olfactory dysfunction was a little-recognized and underestimated disorder, distressing to those affected and with few effective treatments available. Prior to the COVID-19 pandemic, olfactory dysfunction was believed to affect approximately 19% of the general population, rising to 40% in those aged over 60 years [1]. The etiologies most commonly reported were sinonasal disease, upper respiratory tract infection, and traumatic brain injury [2].

Since the outbreak of COVID-19, cases of olfactory dysfunction have increased. The most recent estimate for COVID-19–related loss of smell and taste is 50% to 65% of all cases [3,4]. Given that there have been >200 million cases of COVID-19 globally (as of August 13, 2021) [5], approximately 100 million people will have been affected by smell loss. Although many will recover within weeks [6], it is estimated that approximately 10% will have long-term olfactory problems, many of whom will subsequently develop a qualitative olfactory dysfunction [7].

### Qualitative Olfactory Dysfunction

The term *qualitative olfactory dysfunction* covers both parosmia (qualitative distortion in the presence of an odor) and phantosmia (odor experience in the absence of odor).

Parosmia is the triggered (requiring an external stimulus), subjective perception of a qualitatively altered odor identity with a negative hedonic component (almost always unpleasant), and it usually subsides within seconds of the stimulus. It often develops in the early stages of recovery from smell loss, particularly after postinfectious and posttraumatic anosmia (loss of any sense of smell), with onset weeks after the initial insult. Those severely affected by parosmia find many familiar foods intolerable and start to reject food, leading to weight change, anxiety, and in severe cases, clinical depression [8-10]. Parosmia has been reported in 34% of all patients presenting with olfactory disorders (n=392), and the most common etiology is upper respiratory infection, with 56% of those with anosmia progressing to parosmia [11].

Phantosmia often occurs alongside parosmia [12] and is similarly a perception of an unpleasant subjective odor; however, it is not triggered by obvious external odorants. Patients experience many of the same objectionable odors that are perceived by those with parosmia [2], but these sensations can persist for days.

### Role of Social Media

Awareness of smell disorders has undoubtedly increased since the start of the COVID-19 pandemic, after it became recognized worldwide as one of the key symptoms [13]. However, relatively few publications are dedicated to understanding either the pathophysiology of the disease and its impact on the patient. The charity AbScent (Registration No. 1183468 in England and Wales) has provided support for those with such disorders, launching several support groups on Facebook, the first in 2015 [14]. A COVID-19 Smell and Taste Loss group was started in March 2020 [15], and once it became apparent that post–COVID-19 anosmics were also developing parosmia, a group dedicated to those experiencing parosmia and phantosmia was started in June 2020 [16]. This group accumulated >7000 followers by February 2021 and is an important source of information for researchers, providing valuable insight into the nature and progression of the disease. The fact that people are turning to social media for support is evidence that patients are in need of more information to help them understand both the disease and the efficacy of various treatments, as well as finding social interaction and moral support in coping with an often debilitating condition.

### Aims of This Study

The aims of the paper are to qualitatively explore the symptoms and sequalae of postinfectious olfactory dysfunction syndrome, using unstructured and unsolicited threads from social media, and to articulate the perspectives and concerns of patients affected by these debilitating olfactory disorders.

## Methods

### Approach

The use of social media is rarely discussed in the published literature; however, Alanin et al [17] argue strongly that this approach, when driven by the patients’ own perspectives, provides invaluable, unsolicited, and spontaneous data that would not otherwise be retrieved from more structured surveys and questionnaires. It paints a rich picture of the patient journey, avoiding bias arising from the structure of the survey and the generation of artefacts where patients are eager to please. Other biases may be introduced with this method; for example, the sample is not randomized, and no conclusions about incidence or prevalence can be made due to self-selection.

### Source of Data

The findings reported here are taken from posts made in the AbScent Parosmia and Phantosmia Support closed group on Facebook between June 12 and December 14, 2020 [16]. Demographic data are not available, as those joining the group do not provide this information. Conversation within this group is lively, and responses to polls and questions can generate over 300 comments in 24 hours. Although much of the research into the changes in smell and taste during the pandemic has focused on patients who have had confirmed cases of COVID-19, either through positive tests or clinical diagnosis, the information presented here is about recent self-reported changes that have not been confirmed as being related to COVID-19 infection.

This study has been approved by the University of Reading School of Chemistry, Food and Pharmacy Research Ethics Committee (study number 39.2020), and express permission was sought through messaging channels from each person quoted.

### Thematic Analysis

Thematic analysis was conducted by following the approach used by Alanin et al [17]. An inductive approach was used to allow the data to determine the themes, and a flexible reflexive thematic analysis was used to code the threads. The Facebook group moderator (CK) monitored the discussion daily, noting recurring themes, and a second researcher (JP) independently reviewed the posts to identify themes. The themes were reviewed, and after discussion, 7 major themes were identified by consensus and subthemes emerged. Quotes that were deemed to illustrate the major ideas in each theme or subtheme, and for which permission for inclusion had been freely obtained, were selected for presentation (verbatim) in Table S1, Multimedia Appendix 1.

### Content Analysis

To identify the foods most often associated with parosmia, the data from one thread of conversation were collated manually and counted twice. The conversation was prompted by the moderator, who posed the question: “Can you all add here your worst foods for parosmia?” (137 comments). Data were collected during the first 3 months of the study using a simple frequency table to record each food as it was mentioned (Table S2, Multimedia Appendix 1). In terms of text analysis, this is a very small data set, and manual coding was deemed appropriate, although the repeatability and tolerance associated with this method should be borne in mind.

## Results

### Theme 1. “How It Makes Me Feel”

An extensive and concerning theme demonstrated the detrimental effect of parosmia and phantosmia on emotional well-being. Members of the Facebook group reported being sad and miserable (comments 1-5 in Table S1, Multimedia Appendix 1). Some were quite scared and terrified by the prospect of having parosmia (6-9) and found reassurance when they found others in a similar position (10-13). Patients felt alone and isolated (14-15) and were relieved to find the support of the online community. Some were frustrated with a lack of understanding from others (16) and responses from their physicians, who seemed uninterested (17-18) and lacked treatment options. Many reported a decline in their mental health (19-23), and one member was surprised that loss of smell could have such an impact on their mental health (24). Lack of the ability to smell body odor was a worry, as was the inability, from a safety perspective, to smell smoke or gas. Some adjusted to the “new normal” (25), and many were looking for hope (26-28). Although early in the process, some posted about their recovery (29-33), which was usually only partial. Few mentioned complete recovery, but by this stage, these users might have left the group without commenting further.

### Theme 2. Fluctuations

One emerging theme is the fluctuating nature of the symptoms during the recovery process. Often, users experienced partial recovery (hyposmia), and many reported a complete return of a normal sense of smell before the onset of parosmia (34). The duration of symptoms varied, as some had occasional “whiffs” of normality occurring during long stretches of parosmia (35). For others, the changes in intensity and the magnitude of the fluctuations were quite extreme (36-38) and persistent (38), and the severity of parosmia and phantosmia (92-100) could vary daily. Both hormones (38) and tiredness or stress (39) were associated with more severe fluctuations.

### Theme 3. Items That Trigger Parosmic Experiences

Much of the online discussion concerned problematic foods, drinks, or household items, as patients started to recognize and record their own experiences and explore whether the same items elicited the same response in others. Some listed up to 15 to 20 different items, including food, drinks, bleach, cigarettes, and personal care items (shower gel, shampoo, and hand sanitizers). Many mentioned coffee to be one of the worst triggers (40-41). Fried, toasted, or roasted foods were common triggers of distortion (42-44), as were chocolate (45-46) and onions and garlic (47-49); however, these were not universal. Long lists of other foods were provided that included carbohydrates, fruit, vegetables, herbs, spices (50), and even water (51); thus, it was almost impossible to create a list of foods that never triggered parosmic sensations.

Personal care items were frequently mentioned (42-55); many users attributed their parosmia to different brands or ingredients and started exploring alternative products. Toothpaste and mint were frequent offenders (56-59), and there were recommendations to switch to alternative flavored toothpastes (60).

### Theme 4. Defining the Character of Parosmic Distortions

One defining feature of parosmia is the distortion of (familiar) smells, which members had difficulty describing because they could not relate the smell to a previous experience (61-65). Although many descriptions were used to describe “that parosmia smell,” they were often prefixed with “it’s like” in an attempt to describe the associated disgust rather than the smell identity. To many, it was an entirely novel smell. A group of frequently used words seemed to be based on a burnt, chemical, or dirty connotation: burnt cigarettes, burnt rubber, sewage, earthy, dirty, rancid, death and decay, or unpleasant (66-70). However, there was evidence of at least one other type of parosmia smell, with many reporting their triggers as sickly sweet and rotten (71-73). These two different concepts were often attributed to different foods (74-75) but had been used together to describe one parosmia smell (76) or in a progression in which the burnt character appeared first but changed into the sweeter smell after several months (77).

The feeling of disgust and revulsion associated with the distortions (78-79) was clear and could induce vomiting in a handful of cases (80-82). Disgust was not always mentioned explicitly, but many implied their disgust by their choice of words, which were associated with disgust (garbage, sewage, decay, feces).

### Theme 5. The Smell of Feces

This was a recurrent theme in the Facebook group. If perceived at all, the smell of feces was usually more pleasant than expected (83-85) and often took on the same character as food, such as onion and garlic, whether this was a distorted (86) or normal (87) onion and garlic smell. The smell of feces was often described as distorted coffee (88-89) or as a sweet smell (90-91). The striking corollary of this finding is that the hedonic value of these odors was reversed: odors that typically elicit disgust were less objectionable than before, but odors that usually had a positive hedonic value were perceived as disgusting, and the confusion between food smells and bodily waste products could cause additional distress.

### Theme 6. Phantosmia

Many group members confused parosmia and phantosmia (92), not knowing whether what they perceived was real (93). In general, phantosmia was discussed less frequently, even though it caused much anxiety, as the perceived odor could last for days (94), weeks (95), or even months. Similar to parosmia, the descriptions of the smell were cigarette, chemical, burnt and rotting (96-97), or sweet and sickly (99), and the phantosmia was subject to fluctuations (96, 98). An unusual and novel finding was that in some cases, members described it as a triggered reaction (100-101); this was echoed strongly in the AbScent COVID-19 Smell and Taste Loss group, where there was a thread dedicated to phantosmia.

### Theme 7. Tips and Tricks for Survival

Evidently, the members of the group provided significant support for each other (102-103), creating a positive environment with few negative comments. Some posted practical tips to mitigate the impact (104-106), and many were keen to pass on their own experience and provide lists of “safe” foods that cause minimal distortions. In general, it is unsurprising that appetite was lost (107), as bland foods were frequently recommended (“the plainer the better,” 108-109)) and many turned to fresh fruit and vegetables (110), plain carbohydrates (111-112), and dairy products (112-115) as “safe” options. Cooked and roasted foods that tend to be a major source of protein (meat, nuts) were not considered “safe,” and it is concerning that some experienced difficulty finding a palatable source of protein (116-117). Whereas some found acceptable alternatives (Quorn, turkey mince, and protein shakes), others reported diets lacking in anything nutritious (118-119) or abstained from food completely (120), putting themselves at risk of malnutrition.

### Content Analysis

More than 75 different items were mentioned as triggers of a parosmic sensation in a conversation prompted by the moderator. By using our selection criteria (avoiding multi-ingredient foods, focusing on simple ingredients, and combining where necessary), a list of 50 items that trigger distortions, and the frequency with which they were cited, is shown in Table S2. The four major food triggers, each cited >40 times, were coffee, meat, onions, and toothpaste/mint, followed by garlic and eggs, which were cited >20 times. Personal care products such as shower gel, deodorant, soap, shampoo, and hand sanitizer were often triggers, and these were gathered into a single category and were mentioned >30 times.

## Discussion

### Principal Results

A total of 7 themes emerged from our analysis, as outlined below:

- Theme 1 confirms that the negative emotional and mental health impacts of smell dysfunction, already described in the literature [8,9,18,19], are prominent in those experiencing parosmia and phantosmia.
- In Theme 2, we uncover the fluctuating nature and duration of the symptoms of parosmia and phantosmia during the recovery process.
- In Theme 3, we identify a group of the most common items (coffee, meat, onion, and toothpaste) that trigger distortions, and this was supported by a quantitative content analysis of the threads.
- In Theme 4, we demonstrate how difficult it is for those with parosmia to describe their perception of distorted items, and that most describe their hedonic reaction to the distortions rather than describing the characteristics of the smell.
- In Theme 5, we collate people’s responses to fecal smells. We observe a hedonic reversal in that fecal smells are often reported to be more pleasant (or less unpleasant) than before olfactory loss or change.
- In Theme 6, the focus is on phantosmia, or distortions that are untriggered; however, we observe what we believe is a novel symptom, which we term “olfactory perseveration”—a triggered, identifiable, and usually unpleasant olfactory percept that persists, sometimes for days, in the absence of an ongoing stimulus.
- Theme 7 identifies tips and tricks for survival as provided by the members of the group.

In the discussion that follows, we have synthesized the thematic findings to discuss the emotional and clinical manifestations of qualitative disorders, common triggers, and coping strategies.

### Emotional Manifestations

**Figure 1:**
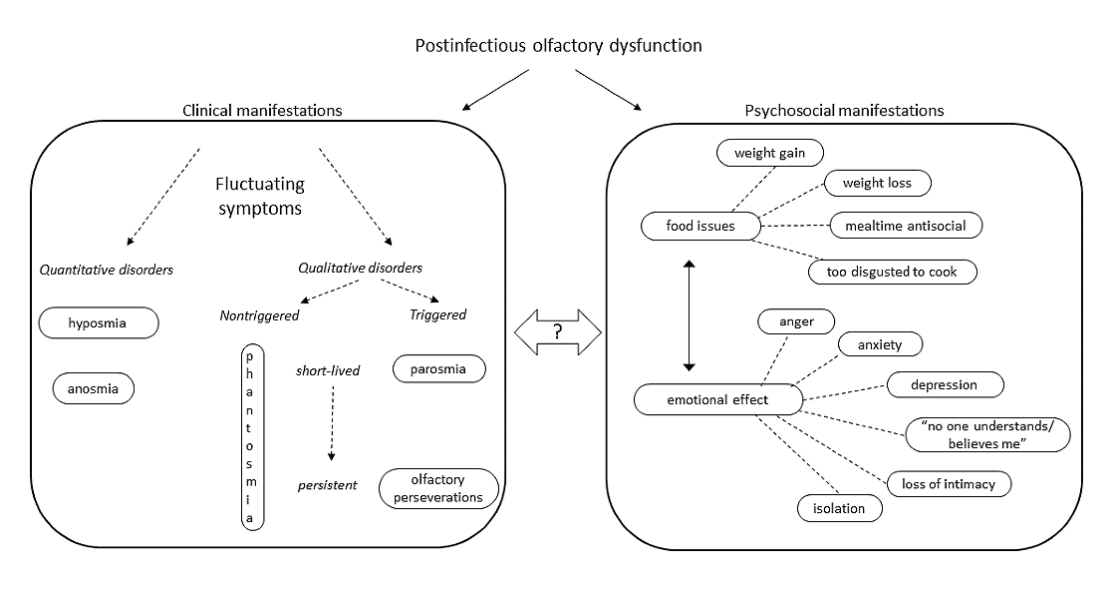
Clinical and psychosocial manifestations of olfactory dysfunction, incorporating conclusions from Burges Watson et al [19].

The emotional and mental health impacts of smell disorders was a major topic of discussion in the group. Figure 1 summarizes the psychosocial manifestations, incorporating observations on food issues from Burges Watson et al [19] showing the interdependence of food issues and emotional effects. Smell loss or change is acutely felt by those suddenly confronted with the way their experience of the world is altered in terms of a lack of pleasure in eating and the absence of reassuring smells of familiar people and places. Many feel socially isolated and go on to experience long-lasting depression.

### Clinical Manifestations

Accounts of clinical manifestations are summarized in Figure 1, which shows an undefined yet complex and fluctuating relationship between different olfactory disorders (Theme 2). Contrary to what we have learned anecdotally from non–COVID-19 incidences of parosmia, posts in this recent group indicate that parosmia can be preceded by the almost complete return of a normal sense of smell. However, it was demonstrated recently that there is a mismatch between subjective self-reported olfactory function and objective olfactory tests: more than half of 112 patients reporting a normal sense of smell were found to have a quantitative loss of sense of smell, of whom 6 were anosmic [20]. During further progression of the disease, experiences of the symptoms of olfactory disorders vary in intensity and duration (prolonged periods or short whiffs), giving episodes of different qualitative olfactory disorders and intermittent returns to anosmia or even to what is perceived as normal olfactory function. These fluctuations have no obvious basis or sequence, can occur in series or in parallel, and vary from case to case. Given the close association between olfactory function and mental well-being [8], it is conceivable that fluctuations may be influenced by emotional status.

What was surprising in Theme 6 was the elucidation of what we believe to be a novel symptom: “smell lock” or olfactory perseveration. A search of the literature finds only one mention of this phrase, in a paper on olfactory hallucinations in Alzheimer disease [21], where it was not further described. We propose a definition of this symptom as a triggered, identifiable, usually unpleasant olfactory percept that persists in the absence of an ongoing stimulus. We suspect that it is often confused with phantosmia and is relatively common in postinfectious smell loss syndrome. It is distinct from phantosmia in that it is triggered by a recognizable stimulus and remains identifiable as the smell of that trigger while persisting long after the original stimulus is removed from the surrounding environment. Describing novel symptoms such as this both increases our understanding of patient experience and guides further research efforts.

### Common Triggers

The wide range of trigger foods and personal care products identified in Theme 3 is consistent with a survey of 725 patients conducted by Keller and Malaspina [2]. This study also reported coffee as an “efficient” trigger, and it was often mentioned together with chocolate. Meat, coffee, and cocoa represent some of the most complex aroma profiles, as the exposure of the combination of sugars, amino acids, and fats to high temperatures (coffee roasting, roasting of cocoa nibs, roasting meat) often produces a rich mix of aroma compounds. Because these and other cooked foods listed (fried foods, peanut butter, bacon, and toast) are frequent triggers that are rarely reported as “safe foods,” it is reasonable to suggest that they may have aroma compounds in common that trigger parosmia. Onion and garlic are mentioned far less frequently in the Keller and Malaspina study but are major triggers of parosmic distortions in this study. Both contain a variety of potent sulfur compounds, which we propose may be responsible for initiating the experience of parosmia. Many unheated foods are also triggers (bell pepper, citrus, apple, cucumber, and banana); therefore, parosmia is not simply associated with cooked foods. Mint and toothpaste also seem to be powerful triggers, although it is not yet clear whether this may also be due to stimulation of the trigeminal nerve. Personal care products are objectionable to many, but these are very brand dependent and subject to the ingredients, particularly the choice of essential oils and flavorings used by different manufacturers.

In Theme 5, we find that the smell of feces takes on the character of other distorted foods, suggesting that the strongly objectionable odors typically associated with feces are not being perceived. There is a curious switching in hedonic valence between odors that are usually objectionable and those that are usually highly desirable: it seems that “fair is foul, and foul is fair” in parosmia.

### Coping Strategies

Advice on food is crucial from nutritional, hedonic, and emotional perspectives (Theme 7). Avoidance of the top triggers (coffee, meat, eggs, onion, garlic, and toothpaste) and most roasted or baked foods makes sense; however, this may lead to nutritional deficiencies, especially in those who rely on meat and eggs as their main sources of protein. Furthermore, avoidance of triggers may hinder the adaptation process that occurs, albeit very slowly. This is one area where further research would be beneficial. It is far better for those afflicted to develop coping strategies for unpalatable foods. This could involve minimizing thermal load; boiling or steaming rather than roasting or frying; and minimizing flavor release by consumption of foods that are chilled or at room temperature. What is clear is that every person is different, and people need to experiment to find a varied diet with the right balance of nutrition and reward.

### Conclusions

Qualitative methods are sometimes perceived as being less rigorous than quantitative methods; however, we have shown how valuable such methods can be in both understanding patients’ lived experiences and constraining hypotheses for quantitative investigation. By collation and interpretation of themes derived from social media, we have articulated the concerns and experiences of patients and gained a better understanding of qualitative olfactory dysfunction and the stimuli that trigger the responses of disgust, learning over time how the disease progresses and how it affects eating behavior, social interactions, and mental health. Forums such as the AbScent Facebook group provide much-needed moral support, where members immediately feel connected, listened to, and relieved to know that they are not alone. This itself can lift spirits, representing an important first step in coming to terms with what is, for many, a relatively long-term condition. The value of patient-centered research should not be underestimated. This qualitative research, based on thematic analysis of unsolicited social media posts, provides the framework for ongoing investigation and therapeutic intervention in smell disorders, shedding further light on the impact of these problems and guiding the direction of further research.

## Appendix

Multimedia Appendix 1 Supplementary tables.
