# Supplementary material for: Patients’ Perspectives on Qualitative Olfactory Dysfunction: Thematic Analysis of Social Media Posts
Source: JMIR Form Res. 2021 Dec 14;5(12):e29086. doi: 10.2196/29086 (PMC8673716; doi:10.2196/29086)
Supplement: Multimedia Appendix 1 [file formative_v5i12e29086_app1.docx]

**Table S1** Extracts (verbatim) from the AbScent Parosmia and Phantosmia Support Facebook group

|  |  |  |  |
| --- | --- | --- | --- |
| **Theme and Subtheme** | | **No** | **Quotes from the AbScent Parosmia and Phantosmia Support Facebook group** |
|  |  |  |  |
|  |  |  |  |
| **Theme 1: Parosmia – how it makes me feel** | | | |
|  | sad | 1 | I feel sad and depressed and distanced to the world as it used to be. Not being able to smell the most mundane things, like the rain or my boyfriends perfume. Not being able to participate socially like i used to. |
|  |  | 2 | I just feel sad about all of this and am worried |
|  | miserable | 3 | I am in the peak stage and feel miserable, especially because I fear this is how my life will be forever and I am not sure I can cope. |
|  |  | 4 | I feel like I'm losing myself as I was such a foody but now I feel I'm only eating to survive. |
|  |  | 5 | It is so miserable and it makes you feel so low experiencing this |
|  | scared | 6 | made me cry I was so scared |
|  |  | 7 | I was very glad to find this group because although what we are experiencing is awful, it helped me to feel less scared about what is happening |
|  | terrified | 8 | This is terrifying, how long does this stage last |
|  |  | 9 | the loss of my smell and taste has affected almost every part of my life |
|  | not alone | 10 | I have literally only just found out that this isn't just me!! |
|  |  | 11 | I have found some comfort in reading these posts as it reminds me I’m not alone. |
|  |  | 12 | but I am so relieved this is actually a thing and it hasn't just happened to me |
|  |  | 13 | it is such a relief knowing you aren't alone isn't it! |
|  | alone | 14 | I feel lost, alone, defeated depressed! |
|  |  | 15 | I coped with anosmia for 3 months but the parosmia had me seeking out this group. It’s distressing and isolating. |
|  | frustrated | 16 | People who are not suffering just do not understand the hell we are going through. |
|  |  | 17 | so when I phoned the doctors after a few weeks they didn't want to know |
|  |  | 18 | My GP doesnt understand my problem. |
|  | mental health | 19 | parosmia started in the beginning of September, and it has taken a toll on my mental health. |
|  |  | 20 | Really feel like no one understands the impact it’s had on my mental health. |
|  |  | 21 | I’m really struggling and it’s so hard when no one around you understands , the thought of it going on this bad for months on end is unimaginable |
|  |  | 22 | I was crying everyday, and finally called my doctor for some anti-depressants |
|  |  | 23 | It is so miserable and it makes you feel so low experiencing this |
|  |  | 24 | I wouldn’t have expected smell to have this big of an impact on mental health. |
|  | adjusting | 25 | I have noticed some days are worse than others, but I’m not crying as much about it anymore and my anxiety levels aren’t quite as high as I am adjusting to this new normal. |
|  | hope | 26 | I would like hear stories on how to cope and successful recoveries. |
|  |  | 27 | Interested to hear other peoples coping techniques. |
|  |  | 28 | Thanks for lifting my spirits and giving me hope!! |
|  | recovery | 29 | A few weeks ago my parosmia has improved and is not as strong as it was before, |
|  |  | 30 | I’m not better but can see improvement |
|  |  | 31 | I'm almost recovered now! I had covid in March and the foul smell/taste started around June and was horrific for about a week, bad but not as bad for another fortnight and then slowly started improving. I still have a few things that trigger it but now it's nowhere near as extreme |
|  |  | 32 | I hope I can give you some hope, I lost my smell and taste 11th March and parosmia came 2 months later. It was horrendous for a while, I lived off very bland food. Then it gradually became more tolerable and now I can actually taste some things almost normally again |
|  |  | 33 | I'd still say I'm about 80% recovered |
| **Theme 2: Fluctuations** | | | |
|  |  | 34 | Anosmia for about 1 week in March. Smell and taste returned to somewhere near normal. In June 2020, parosmia began. |
|  |  | 35 | Parosmia is same at 8 months but very occasional, normal whiffs |
|  |  | 36 | Lost my smell and taste in April, some smell/taste came back...then crazy Parosmia since August. I think it’s getting better and then wham it’s terrible again! I am trying a more positive attitude and finding it helps... |
|  |  | 37 | I actually thought the Parosmia was lessening…... but it’s back just even with smell of flowers , tea , squash |
|  |  | 38 | The fluctuations are really annoying, I find some days it’s normal parosmia only (stale,sour but sweet smell) but other days there will be some enhanced “bad smells” and phantosmia combined where I can’t escape the smell. I wonder if hormones contribute to this and maybe explain fluctuations through the month? I have found my fluctuations are getting much less as time goes on and some foods taste almost normal. |
|  |  | 39 | There absolutely is fluctuations in parosmia which seems really strange. I'd say either being tired or stressed seems to make the taste worse. |
| **Theme 3: Items that trigger parosmia** | | | |
|  | coffee | 40 | Coffee, without a shadow of a doubt, is the worst. |
|  |  | 41 | The absolute worse is freshly brewing coffee (the grounds) |
|  | cooked food | 42 | anything toasted (bread, nuts...) |
|  |  | 43 | anything deep fried in oil |
|  |  | 44 | Anything toasted or roasted turns to that awful smell |
|  | chocolate | 45 | Coffee (obviously), anything chocolate, cola, chicken, sausage, onions, garlic, most chips, toothpaste (& almost any minty things), most "fruity" alcoholic beverages, most cleaning products (except unscented or all natural products). |
|  |  | 46 | I have chocolate cake to make now and last week it tasted/smelt of poo |
|  | onions | 47 | Anything containing the slightest bit of onion or garlic are the worst for me and I literally cannot eat it without gagging. |
|  |  | 48 | For me the worst is onion |
|  | the worst | 49 | Egg, garlic, onion, all meat, chocolate Coffee |
|  | fruit and veg | 50 | Absolutely can't bear Onions, Garlic, Potato in all forms, mint, chocolate, coffee, any berries, Salmon, all meat, most bread, Green Peppers, Cereals, toothpaste, peas, Carrots, tomatoes & Celery so not v much at all. |
|  | water | 51 | I could not identify the trigger. I then realised that it was caused by water! …. Tap Water smells and tastes unpleasant (bottled water is fine). |
|  | personal care | 52 | Anything chemical like shower gel to washing and cleaning products all have one smell,and even that is horrible |
|  |  | 53 | Bathroom stuff - deodorant, shampoo, moisturiser etc.... most smell horrid, |
|  |  | 54 | Even brushing my teeth or taking a shower (when using shampoo or shower gel) is horrible. EVERYTHING is permeated by this terrible situation. |
|  |  | 55 | gave my baby daughter a bath and the smell of her soap was just awful and made me feel sick |
|  | toothpaste | 56 | My worst tastes are mint toothpaste, mint chewing gum…. |
|  |  | 57 | Toothpaste tastes vile, makes me gag. |
|  |  | 58 | Anything with mint (toothpaste, ice cream, mojitos...) |
|  |  | 59 | mint of any sort( toothpaste is like cleaning teeth with petrol!) |
|  |  | 60 | I had no luck with cinnamon toothpaste, but I found a vanilla chai toothpaste that does the trick! |
| **Theme 4: Describing the distortions perceived** | | | |
|  | indescribable | 61 | I have never smelt/ tasted this specific smell so there’s nothing I can describe it as. |
|  |  | 62 | Very hard to describe... it is a new smell... I have never smelled that in my normal life... I tried to say what it is like.... but it is like nothing.... |
|  |  | 63 | I’ve never smelt this before so I find it hard to describe. |
|  |  | 64 | Like everyone has said it’s impossible to find the words! |
|  | funky | 65 | My word for it is "funky". It's not like anything else but I describe it as a sweet, sickly, sour odour. |
|  | burnt | 66 | everything smells like Burnt cigarette and cleaning solutions |
|  | sewage | 67 | There was one week in particular in July that was horrific, everything smelled and tasted like raw sewage and trying to eat made me vomit. |
|  | dirty | 68 | A taste/smell I have never had before. Dirty, earthy, burnt |
|  | unpleasant | 69 | It carries a deep woody , burnt unpleasant smell. |
|  | decay | 70 | Rancid, cloying, death |
|  | sickly sweet | 71 | Most taste/smell awful either Vomit with a side of bin juice or acrid smoke with a side of v sickly sweet chemicals!! |
|  |  | 72 | Sickly, rotten, sweet, overpowering old rubbish!!! |
|  |  | 73 | it's a relief not to smell the sweet chemical sickly smell so much |
|  | binary | 74 | Some smells are like rotten melon (shampoo, deodorant) and some are more smoky (coke, shaving cream, parsley), but I think they are related. |
|  |  | 75 | Sometimes like bile and sometimes like rotten garlic |
|  |  | 76 | Each thing that smells bad to me …..... all have the same chemical, rotted burnt meat, with a touch of sweet smell to them |
|  |  | 77 | At first everything was like dead body/burning. Now everything has a sweet, chemical, nutty smell at 8 months in |
|  | disgust | 78 | it is new and disgusting |
|  |  | 79 | It's foul. Makes you screw your face up. It's disgusting. |
|  | vomit-inducing | 80 | I was entering Tesco and whatever it was that triggered the smell I’m not sure but it made me vomit there and then |
|  |  | 81 | but the smells are nausea inducing. |
|  | making me retch | 82 | Chicken is making me wretch. Getting more and more anxious about certain foods and especially my family's reaction to me eating or not eating things. |
| **Theme 5: The smell of feces** | | | |
|  | no longer bad | 83 | They don’t smell as bad as they probably should |
|  |  | 84 | They don’t smell too bad! |
|  |  | 85 | Poo smells a distinct odour but not like it used to and nowhere near as horrible |
|  | parosmia smell | 86 | Feces, urine, body odor & deodorant all had the same nauseating parosmia smell for me (same as onion & garlic) |
|  | onions | 87 | Wee and poo smell strong and horrendously like onion/garlic. |
|  | coffee | 88 | I relate the poop/fart with new weird smell of the coffee |
|  |  | 89 | It’s faint though, the smell is wrong and weirdly it smells the same as coffee and cigarette smoke |
|  | fruity sweet | 90 | they don't smell offensive too me when I can smell them, often I can't smell it at all. But the smell they have is the same as coffee, peanut butter and citrus fruits weirdly. |
|  | chemical | 91 | More recently they smell of a sweet chemical smell, |
| **Theme 6: Phantosmia** | | |  |
|  | confusion | 92 | I’m a bit confused, as I think I have a mix of Parosmia and smell/taste lock |
|  |  | 93 | With phantosmia, I don't know what's real. |
|  | duration | 94 | At first I had a relatively constant smell of burning plastic/tyres which lasted for about three days, then subsided. |
|  |  | 95 | I have been experiencing phantosmia on and off for the last few weeks, which would be about 11/12 weeks after losing my smell. |
|  | descriptions | 96 | Mine comes and goes, and about 95% of the time it's the smell of cigarette smoke. |
|  |  | 97 | Cigarette smoke, noxious chemicals, burning smells, rotting smells . |
|  | sweet | 98 | It definitely comes and goes, sometimes very intense to the point of being slightly nausea-inducing, at other times barely perceptible. |
|  |  | 99 | I have had periods of a quite sweet smell |
|  | triggered | 100 | My personal experience is my phantosmia is mostly manageable until an actual smell triggers it. I will than smell rotten veggies for hours and hours |
|  |  | 101 | I have this when my husband cooks meat, in particular chicken. The smell stays in my nose for days and it is horrid. |
| **Theme 7: Tips and tricks for survival** | | | |
|  | support | 102 | We are all right there with you!! |
|  |  | 103 | Knowing I have a safe place to vent, and grieve and a place to find strength when I am weak is the best therapy. Hang in there.... |
|  | practical tips | 104 | Just wanted to share a little tip for those feeling really nauseous from parosmia. Chamomile tea helped me so much! Not only it tasted as it should but it really helped soothing my stomach when even water was making me sick. Also - it will improve! Just hang in there. |
|  |  | 105 | Please if you’re struggling with smells and eating try to use a nose clip, like those used to swim. |
|  |  | 106 | Tip of the day for all you coffee lovers!!!! Iced coffee through a straw tastes normal! |
|  | appetite | 107 | It's nauseating, has stripped me of appetite and is really hitting my (already fragile) mood. |
|  | plain foods | 108 | Seems to me that the plainer the better lightly poached fish like cod, haddock or prawns, nothing strong tasting, and boiled potatoes or rice. |
|  |  | 109 | The plainer the better at the minute |
|  | fruit and veg | 110 | Avocado, baby sweetcorn, bamboo shoots, beansprouts, broccoli, brown bread (not toasted), cauliflower, chilli, courgette, coconut/coconut milk, grapes, green beans, kale, lettuce, mange tout, nectarines, nuts, quinoa, radish, raspberries, rice, runner beans, sesame seeds, strawberries, sweet potato, tofu, raw tomatoes, water chestnuts, soya yoghurt |
|  | carbohydrate | 111 | Latest discovery: rice pudding tastes ok!! |
|  |  | 112 | Plain potatoes, yoghurt and ice cream were my only safe foods |
|  | dairy | 113 | Cheese has been a hero throughout |
|  |  | 114 | I can only eat bread, cheese, natrural yogurt, just basic bland non smelling food. |
|  |  | 115 | muesli with fruit and greek yoghurt |
|  | proteins | 116 | I cant seem to find any protein that is tolerable. |
|  |  | 117 | I am pregnant and struggling to get enough protein and also vegetables in bc of parosmia |
|  | poor nutrition | 118 | After eating little else except bread, cheese, chips and cake since June |
|  |  | 119 | I'm surviving on milkybar buttons and jammie dodger biscuits at the moment as they are the only 2 things that truly taste as they should |
|  |  | 120 | Today has been a really bad day for me (where I feel everything that passes my nose is unbearable, even my freshly washed bedsheets) I've found myself just not eating at all. |
|  |  |  |  |

**Table S2** Count of items triggering parosmic sensations taken from 137 comments retrieved from the AbScent Parosmia and Phantosmia Support Facebook group between 8^th^ August and 30^th^ September 2020

| **Cooked foods** |  | **Fruits, vegetables,** |  | **Alliums/Brassica** |  | **Drinks** |  |
| --- | --- | --- | --- | --- | --- | --- | --- |
| **Maillard** |  | **herbs and spices** |  |  |  |  |  |
|  |  |  |  |  |  |  |  |
| meat (chicken, beef, lamb) | 45 | banana | 14 | onion | 47 | wine red and white | 14 |
| coffee | 42 | citrus | 13 | garlic | 27 | cola | 11 |
| eggs | 28 | peppers | 6 | mustard | 3 | water | 1 |
| chocolate | 14 | cucumber | 6 | rocket | 2 |  |  |
| fried foods | 11 | coriander/cilantro | 6 | broccoli | 1 | **Non-food items** |  |
| peanuts/peanut butter | 9 | berries | 5 |  |  | toothpaste/mint | 43 |
| bread | 7 | strawberries | 4 | **Dairy** |  | personal care products | 30 |
| bacon | 6 | celery | 3 | butter | 3 | body odours | 5 |
| toast | 5 | jalapenos | 2 | cheese | 3 | cigarette smoke | 4 |
| tomato products | 4 | pineapple | 2 | yogurt | 2 | bleach | 4 |
| oats/porridge | 3 | pesto | 2 | milk | 1 | petfood | 3 |
| soy sauce | 2 | watermelon | 2 |  |  | petrol | 2 |
| marmite | 2 | spices | 2 | **Flavoured products** | |  |  |
| popcorn | 1 | parsley | 1 | sweets | 3 |  |  |
|  |  | peaches | 1 | crisp flavours | 3 |  |  |
|  |  | spinach | 1 | vanilla | 3 |  |  |
|  |  | basil | 1 |  |  |  |  |
|  |  | ginger | 1 |  |  |  |  |
|  |  | apple | 1 |  |  |  |  |
